# Grip strength measurements at two different wrist extension positions in chronic lateral epicondylitis-comparison of involved vs. uninvolved side in athletes and non athletes: a case-control study

**DOI:** 10.1186/1758-2555-2-22

**Published:** 2010-09-07

**Authors:** Arti S Bhargava, Charu Eapen, Senthil P Kumar

**Affiliations:** 1 Ajanta Hospital and IVF Center, Alambagh, Lucknow, Uttar Pradesh India; 2 Department of Physiotherapy, Kasturba Medical College (Manipal University), Mangalore, Karnataka, India

## Abstract

**Background:**

Lateral epicondylitis is a common sports injury of the elbow caused due to altered muscle activation during repetitive wrist extension in many athletic and non-athletic endeavours. The amount of muscle activity and timing of contraction eventually is directly dependent upon joint position during the activity. The purpose of our study was to compare the grip strength in athletes with lateral epicondylalgia in two different wrist extension positions and compare them between involved and uninvolved sides of athletes and non-athletes.

**Methods:**

An assessor-blinded case-control study of eight athletes and twenty-two non-athletes was done. The grip strength was measured using JAMAR^® ^hand dynamometer in kilograms-force at 15 degrees (slightly extended) and 35 degrees (moderately extended) wrist extension positions (maintained by wrist splints) on both involved and uninvolved sides of athletes and non-athletes with unilateral lateral epicondylitis of atleast 3 months duration. Their pain was to be elicited with local tenderness and two of three tests being positive- Cozen's, Mill's manoeuvre, resisted middle finger extension tests. For comparisons of grip strength, Wilcoxon signed rank test was used for within-group comparison (between 15 and 35 degrees wrist extension positions) and Mann-Whitney U test was used for between-group (athletes vs. non-athletes) comparisons at 95% confidence interval and were done using SPSS 11.5 for Windows.

**Results:**

Statistically significant greater grip strength was found in 15 degrees (27.75 ± 4.2 kgms in athletes; 16.45 ± 4.2 kgms in non-athletes) wrist extension than at 35 degrees (25.25 ± 3.53 kgm in athletes and 14.18 ± 3.53 kgm in non-athletes). The athletes had greater grip strength than non-athletes in each of test positions (11.3 kgm at 15 degrees and 11.07 kgm at 35 degrees) measured. There was also a significant difference between involved and uninvolved sides' grip strength at both wrist positions (4.44 ± .95 kgm at 15 degrees and 4.44 ± .86 kgm in 35 degrees) which was significant (p < .05) only in non-athletes.

**Conclusion:**

The grip strength was greater in 15 degrees wrist extension position and this position could then be used in athletes with lateral epicondylalgia for grip strength assessment and designing wrist splint in this population.

## Background

The term 'tennis elbow' was introduced in 1880's, also known as lateral epicondylitis or lateral epicondylalgia [1]. It is the most common source of elbow pain in the general population [2]. It is a soft tissue condition frequently associated with overuse injury, primarily occurring at the aponeurosis of the common extensor origin at the elbow. The common complaints of the individual are pain during wrist extension which is localized to the common extensor origin and decreased grip strength, both of which may affect the activities if daily living [3]. Lateral epicondylitis or epicondylalgia is usually caused by repetitive wrist extension that leads to an overuse injury, followed by micro-tearing of Extensor Carpi Radialis Brevis (ECRB) and occasionally the Extensor Digitorum Communis (EDC) Muscle and Extensor Carpi Radialis Longus (ECRL) muscle [2,4,5]. Examination reveals pain with passive wrist flexion and active and resisted wrist extension. Tenderness is located 1 to 2 cm distal to the lateral epicondyle [6].

Lateral epicondylitis is associated with many athletic and non-athletic endeavours [7]. The annual incidence of lateral epicondylitis is 1% to 3% in the general population. The tennis players account for less than 5% of the population, and exhibit 40% to 50% chance of having lateral epicondylitis at some point in time [8]. Although rarely seen in the elite players 50% of the recreational players can expect to experience this condition at some point of their playing lifetime [9]. Lateral epicondylitis is a condition that primarily occurs in the recreational tennis players [10]. The non-athletic population accounts for 35% to 64% of the population affected by lateral epicondylitis [11].

The force overload implicated in lateral epicondylitis is attributed to the repetitive strong synergistic and fixator role played by the wrist extensors during gripping [2,3]. Gripping activates the flexor muscles thereby creating a flexion moment at the wrist joint and as a result the extensor muscles are co-activated, producing an extension moment that stabilizes the wrist joint.

Recent electromyographic studies have supported the concept that the wrist extensors play a key role in gripping and that ECRB, EDC, ECRL muscles are all activated during gripping [2]. It has been concluded that application of an external wrist extension force reduces EMG activity of the wrist extensors muscles during gripping in healthy volunteers [2].

Thus, wrist extensors play an important role in maintaining wrist in extension and their affection in lateral epicondylitis may hamper the activities of daily living. In 1980, WHO classified lateral epicondylitis as a disability as it often limits the work capacity [6]. It is widely accepted that grip strength provides an objective index of the functional integrity of the upper extremity [7-12]. In clinical setting, grip strength is commonly evaluated by an instrument called dynamometer, which measures static grip strength and is widely accepted to measure the grip strength [13,14].

A number of studies have been done to report the influence of elbow positions on grip strength in subjects with lateral epicondylitis [15-17]. At the time the idea of the study was conceived no study could be retrieved which showed the effect of wrist extension position on grip strength in chronic lateral epicondylitis or one which gives an objective comparison of the grip strength between athlete and non-athlete subjects following chronic lateral epicondylitis. Hence we decided to do a study to determine the grip strength at two different wrist extension positions comprising of 15° and 35° in chronic lateral epicondylitis and to compare the grip strength between the athlete and non-athlete subjects with chronic lateral epicondylitis. This would help in finding the most optimum position in the assessment and treatment of patients with lateral epicondylitis.

## Methods

### Study design

This was a case - control study. All the adult subjects with chronic lateral epicondylitis referred to Physiotherapy department in a multispecialty teaching hospital of a University were included in the study.

### Ethical clearance

The study was approved by the Institutional Scientific Ethics Committee of Manipal University, Manipal and was registered in Clinical Trials Registry-India: UTRN 060144369-190420102025203.

### Participant requirement

Inclusion criteria were pain and tenderness over the lateral epicondyle, at least 2 out of 3 tests for lateral epicondylitis including Cozen's test, Mill's maneuver, or resisted middle finger extension was positive, minimum duration of three months, willing to participate in the study, and ability to comprehend the instructions given by the tester. Exclusion criteria included all the subjects with history of trauma, fracture, surgery, or other medical and non-medical interventions to elbow, bilateral symptoms, polyarthritis, upper quadrant neuromusculoskeletal disorders that might affect grip strength.

### Participant selection

Athlete subjects were those engaged in recreational sports involving tennis or badminton which requires repetitive use of wrist extension position. Non athletes were subjects who were not engaged in any kind of sports activity involving the use of upper extremity. The athletes were recruited from students and staff of the institution while non-athletes involved patients referred for physiotherapy treatment by a medical practitioner. After giving them a detailed explanation about the purpose of the study and its clinical significance a written informed consent was obtained from all the subjects.

### Testing procedure

**Figure 1:**
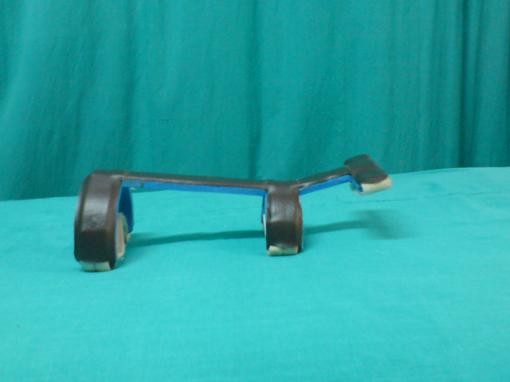
**Dorsal forearm splint for maintaining 15 degrees wrist extension**.

**Figure 2:**
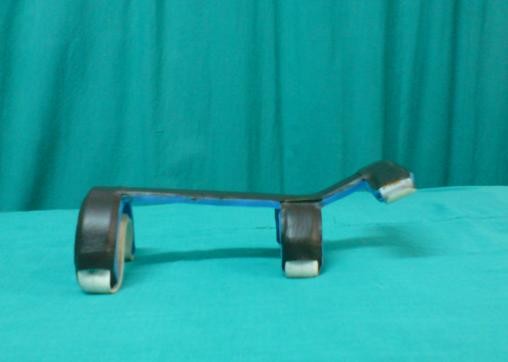
**Dorsal forearm splint for maintaining 35 degrees wrist extension**.

The test was conducted during a single session. After a verbal description of the test procedure, the method of testing was demonstrated to the subject. For each of the tests of grip strength, the standard position recommended by the American Society of Hand Therapists (ASHT) was administered. For performing the test, subjects were seated on a high plinth without supporting the forearms. The shoulder was kept in adduction and neutral rotation; elbow flexed at 90° forearm in neutral position; The wrist positions were kept static by the use of an external support .15° of wrist position was first tested with the splint, followed by 35° (Figures 1, 2). For standardization, the handle of the Jamar^® ^dynamometer was kept at the second handle position.

### Structure of the wrist splint(s)

The experimental splint used for this study was a fabricated dorsal forearm support-wrist extension splint, with dorsal metacarpal bar and ventral Velcro straps to fasten it to the forearm. Two splints were used- one of 15 degrees wrist extension and other of 35 degrees wrist extension (refer figure-1 and figure-2).

### Selection of side for grip strength testing

The first side (involved or uninvolved) to be tested was chosen randomly by a -toss of a coin- method. To ensure that equal number of subjects was tested first on either of their tested side, block randomization was used for further sub-grouping under athlete or non-athlete group. The allocation method was concealed from the primary investigator (tester-1) by the use of sequentially-numbered sealed opaque envelopes.

### Outcome measures

**Figure 3:**
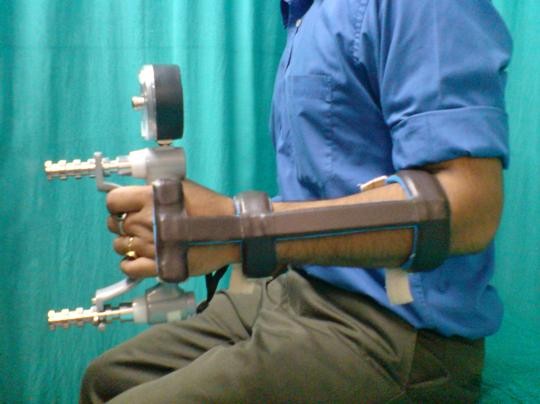
**Person performing gripping action on a Jamar^® ^dynamometer with the splint in position for grip strength measurement**.

Specific instructions were given to each subject before the test. They were asked to "squeeze" the dynamometer as hard as possible, and to hold the position for 5 seconds (Figure-3). No verbal encouragements were given during the test. None of the subjects complained of any discomfort with splint during testing. Grip strength readings were recorded in kilograms. Three trials were performed at each of the15° and 35° of test positions for the uninvolved and involved extremity and the three values were recorded. Each measurement was repeated 3 times. A minimum of 1 minute rest period was allowed between efforts on the same side to minimize the effects of fatigue. To ensure minimized effects of repeated testing on fatigue in performance, random order of testing each of the two positions was done and this was selected by block randomization. The dynamometer was reset to zero prior to each reading. The grip strength was recorded in kilograms-force (kgf).

### Data collection

The readings were taken by another blinded observer and the primary investigator was not aware of the subject's grip strength during the testing.

### Data analysis

Sample size estimation was done using a minimum clinically important difference of 2.0 ± 1.2 kgm (mean ± SD) in grip strength measurements at 80% power and alpha (type-1 error) at 5% level, to be 24. We had an estimated prevalence of athletes (elite and recreational combined) to be one-third of total population. Hence we need to take the study population in 1:3 ratio for athletes: non-athletes. Eight athletes and 16 non-athletes would be statistically sufficient thereby requiring a sample size of 24. There was no anticipated loss to follow-up since the design was a cross-sectional one.

The within-group analysis (comparison between involved and uninvolved sides at both 15 and 35 degrees) was done using Wilcoxon signed-rank test and between-group comparison (comparison between athletes and non-athletes) for grip strength was analyzed using Mann Whitney U-test using the SPSS 11.5 for windows software. The statistical significance was set at p ≤ 0.05.

## Results

30 subjects volunteered for the study (males n = 13; females n = 17). The athlete group consisted of 8 male subjects and the non- athlete group had 22 subjects (males n = 5; females n = 17). The hand dominance of all the participants was right side. Among the athlete group only 1 subject had left side involvement, while in non-athlete group 6 subjects had left side involvement.

### Overall demographic characteristics of the subjects

The number of subjects, subjects' age, duration of symptoms, side of symptoms and side of hand dominance are shown in Table-1.

**Table 1:** Study sample characteristics (number of subjects, age and duration of symptoms) in both the groups

	Athletes	Non-athletes
**Number of persons (male, female)** **Total = 30**.	8 (8,0)	22 (5,17)

Age (in years)*	34.75 ± 7.34	40.5 ± 6.78

**Duration of lateral epicondylalgia symptoms** (in months)*	8.37 ± 9.5	7.6 ± 18.5

**Side of symptoms** **(right/left)**	8/0.	22/0.

**Side of dominance** **(right/left)**	8/0.	22/0.

*- values are mean ± SD.

All subjects complained of discomfort at lateral epicondyle with associated tenderness elicited on palpation during screening examination. 21 subjects reported symptom onset of 3 to 6 months duration, while 7 subjects reported symptom duration of 1 year, and 2 subjects reported symptom duration of 2 years.

### Difference in the grip strength between the involved and un-Involved side at 15°, 35° in athletes and non-athletes

**Figure 4:**
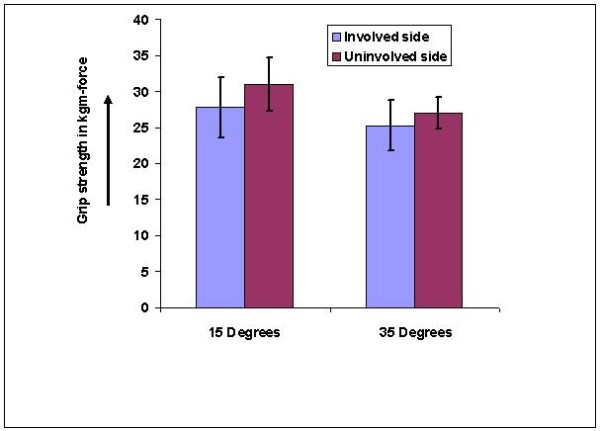
**Bar diagram showing comparison between involved and uninvolved side grip strength in athletes**.

**Figure 5:**
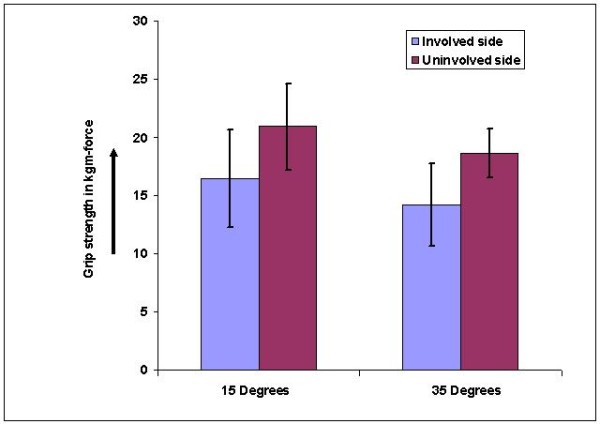
**Bar diagram showing comparison between involved and uninvolved side grip strength in non-athletes**.

There was a significant difference between involved and uninvolved sides' grip strength at both wrist positions (4.44 ± .95 at 15 degrees and 4.44 ± .86 in 35 degrees) which was not significant in athletes (figure-4) but was significant (p < .05) in non-athletes (figure-5).

### Within-group analysis of comparison between grip strength performance at 15 and 35 degrees wrist extension positions for both athletes and non-athletes

Statistically significant greater grip strength was found in 15 degrees (27.75 ± 4.2 in athletes; 16.45 ± 4.2 in non-athletes) wrist extension than at 35 degrees (25.25 ± 3.53 in athletes and 14.18 ± 3.53 in non-athletes).

### Between-group analysis of comparison between athletes and non-athletes for grip strength performance at both 15 and 35 degrees wrist extension positions

The athletes had greater grip strength than non-athletes in each of test positions (11.3 at 15 degrees and 11.07 at 35 degrees) measured. Refer to table-2 for detailed depiction of main results.

**Table 2:** Grip strength comparisons within- and between- groups.

Group	Wrist extension position (in degrees)	**Side of testing**. Symptomatic- S Asymptomatic- AS	Grip strength in pound sq.inch (Mean ± SD)	**Comparison within-group between sides and between positions**. Mean difference (Mean ± SD)	**Comparison between athletes and non-athletes**. Mean difference (Mean ± SD)
**Athletes**	**15°**	**S_1_**	27.75 ± 4.2	S_1_S_2_ 2.5 ± .67	S_1_S_3_ 11.3
			
		**AS_1_**	31 ± 3.7	S_1_AS_1_ 2.25 ± .95	
	
	**35°**	**S_2_**	25.25 ± 3.53	S_2_AS_2_ .75 ± .86	S_2_S_4_ 11.07
			
		**AS_2_**	27 ± 2.13	AS_1_AS_2_ 4 ± 1.57	

**Non-athletes**	**15°**	**S_3_**	16.45 ± 4.2	S_3_S_4_ 2.27 ± .67	AS_1_AS_3_ 10.1
			
		**AS_3_**	20.90 ± 3.7	S_3_AS_3_ 4.44 ± .95	
	
	**35°**	**S_4_**	14.18 ± 3.53	S_4_AS_4_ 4.44 ± .86	AS_2_AS_4_ 8.37
			
		**AS_4_**	18.63 ± 2.13	AS_3_AS_4_ 2.27 ± 1.57	

Within-group comparison was done between symptomatic and asymptomatic sides at both 15 and 35 degrees wrist extension positions and between-group comparison was done between athletes and non-athletes.

Kgm- kilograms-force (units)

*- significant at p value < .05

NS- not statistically significant.

### Difference in the grip strength between 15° and 35° degrees positions of the involved side in athletes and non-athletes

**Figure 6:**
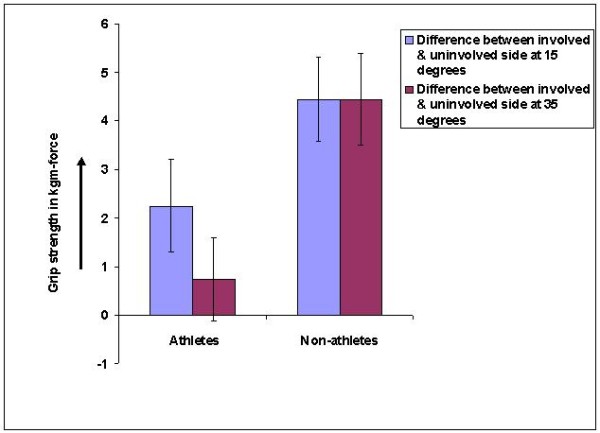
**Bar diagram showing difference in grip strength (involved-uninvolved side) comparison at 15 and 35 degrees in both athletes and non-athletes**.

There was a significant difference between 15 degrees and 35 degrees' grip strength of the involved side (2.5 ± .67 at 15 degrees and .75 ± .86 at 35 degrees) which was significant (p < .05) only in athletes (figure-6 and table-3).

**Table 3:** Grip strength comparisons within- and between- groups with p-values.

Group	Wrist extension position (in degrees)	Grip strength in kgm (Mean ± SD)	**Comparison within-group between positions**. Mean difference kgm (Mean ± SD)	Comparison between-group (athletes and non-athletes) Mean difference kgm (Mean ± SD)
**Athletes**	**15°**	27.75 ± 4.2	2.5 ± .67*	
			
	**35°**	25.25 ± 3.53		.23 (NS)
	
**Non-athletes**	**15°**	16.45 ± 4.2	2.27 ± .67*	
			
	**35°**	14.18 ± 3.53		

kgm- kilograms-force (units)

*- significant at p value < .05

NS- not statistically significant.

## Discussion

This study was done to compare the grip strength at two different wrist extension positions in patients with lateral epicondylitis. It showed that the grip strength is reduced in chronic lateral epicondylitis. The positions of grip strength were selected at 15° as muscle activity of the extensor muscles was found to be less at this position [2]. 35° of wrist extension were found to be the self-selected position for the optimum grip strength. Grip strength was significantly less in any position of deviation from this self-selected position [18]. In our study the grip strength was found to be more at 15° of wrist extension than 35°. This finding is consistent with the EMG study done in healthy subjects where, the highest decrease in muscle activity for ECRB and EDC was at 15° of wrist extension. Thus, at this position the wrist extensors showed a greater advantage for gripping [2]. The increased strength might have resulted from adapting the desired position in patients with lateral epicondylitis. The ECRB and EDC are primarily involved in stabilizing the wrist joint in extension, through their synergistic and fixator role [2]. In chronic lateral epicondylitis these muscles cannot act optimally due to micro-tearing and excessive scarring at their origin [5]. The splint supported the wrist passively in 15° of extension thus, improved their active participation.

Grip strength at 35° of wrist extension position was found to be less. However, this is in contrast to a previous study in healthy subjects, where the optimum position of gripping was found to be at 35° of wrist extension [17]. One of the reasons can be fatigue of wrist extensors as the splint was applied at 15° first. The deviation at the wrist was not taken into account. The influence of other factors, apart from lateral epicondylitis might have also influenced grip strength.

Comparison between the athletes and the non-athletes showed that, the athletes had more grip strength as compared to non-athletes at both the positions. The difference in the grip strength in the involved side between the athletes and the non-athletes was very highly significant (p = 0.001). This was found at both 15° and 35°. The reason could be due to the heterogeneity in the subject characteristics based on gender and type of sports. In the athlete group all were males. The statistics results also showed that the males have higher grip strength. This result is consistent with the studies in normal population. Moreover, the effect of neural adaptation, improved coordination and muscle strength seen in the athletes may have also influenced the results to some extent.

Another interesting fact which was seen was the difference in the grip strength between the involved and the uninvolved side was significant in the non athletes but not the athletes. This could be due to the overall increase muscle strength in the athletes as compared to the non athletes. Hence when compared on both sides the difference was not much in the athlete group.

However a few limitations were noted in the study: (a) less population size so the results cannot be generalized to the whole population. (b) Heterogeneity in the population, athletes consisted of only males and the type of sport was not specific (c) Anthropometric data could not be considered.

Further gender specific studies are needed to prove that the difference in grip strength is dependent on various positions of wrist and then can be compared between the athletes and non-athletes. This study has an important clinical and practical application in designing a wrist extension brace for lateral epicondylitis as 15° approaches the normal functional wrist angle. Though it has been found now that dynamic brace may be a promising intervention for lateral epicondylitis [19] a static brace may still be used in the treatment where the expertise and skill does not exist to make a dynamic one. It may affect the dynamic activity but it will be useful to provide rest and allow the pathological changes in the muscle and tendon to heal [20]. Further it may also guide for functional evaluation of grip strength for subjects with chronic lateral epicondylitis. Maximum grip strength and pain-free grip strength have been used as outcome measures in patients with lateral epicondylitis at the elbow [21].

## Conclusions

In conclusion we can say that the grip strength was more at 15° than at 35° of wrist extension positions in patients with lateral epicondylitis. The athletes have more grip strength than the non-athletes between the two groups. There is a significant difference in the grip strength between the involved and the un-involved side in the non athletes. 15° of wrist extension may be used for testing the grip strength and for designing a brace in patients with lateral epicondylitis.

## Competing interests

The authors declare that they have no competing interests.

## Authors' contributions

All three authors were **equally **involved in substantial contributions to conception and design, acquisition of data, analysis and interpretation of data; and were involved in drafting the manuscript and revising it critically for intellectual content; and gave final approval of the version to be published.
